# Can physical activity maintain normal grades of body mass index and body fat percentage?

**DOI:** 10.4103/0973-6131.53839

**Published:** 2009

**Authors:** C Kesavachandran, V Bihari, N Mathur

**Affiliations:** Epidemiology Section, Indian Institute of Toxicology Research (CSIR), Lucknow, India

**Keywords:** Body fat percentage, body mass index, physical activity

## Abstract

**Background/Aims:**

A cross-sectional study was undertaken on 767 urban male volunteers performing physical activity and 469 age and socioeconomic status matched controls not doing any physical activity from the city limits of North India.

**Materials and Methods:**

Height and weight were recorded for each participant to determine their Body Mass Index (BMI). Body fat percentage and weight was measured using a body fat monitor.

**Results:**

Fifty three percent of the physical activity performers showed normal BMI compared to 49% nonphysical activity performers. Overweight was observed in 43.3% physical activity performers compared to 44.7% nonphysical activity performers. Fifty two percent of physical activity performers had normal body fat percent compared to 48.5% nonphysical activity performers. Low body fat percent was observed in 23.4% physical activity performers compared to 2.7% nonphysical activity performers. High body fat percent was observed in 48.7% nonphysical activity performers compared to 45.8% physical activity performers.

**Conclusions:**

Overall, the study suggested that physical activity alone cannot maintain BMI and body fat percent, but it can reduce the risk of overweight and high body fat percent in the population.

## INTRODUCTION

In the modern society, inactivity or low level of physical activity combined with changes in eating habits are believed to be the main reasons for the increased prevalence of overweight/obesity among children and adolescents.[1] The genetic differences in weight gain among them also make them vulnerable to overweight. As physical activity is an important component in weight control and also associated with other major health benefits,[2] its role in youth health is fundamental. Studies have shown a significant correlation between high physical fitness and a low body fat.[3] Physical activity measured as number of steps per day was inversely correlated to percentage body fat as well as to Body Mass Index (BMI) in adults.[4] However in children, no such correlation was observed.[5] It is therefore difficult to know whether inactivity causes obesity or obesity leads to inactivity. Guidelines for physical activity levels in children and adolescents recommend at least 60 minutes of accumulated moderate to vigorous physical activity per day.[6] There are several difficulties to measure the total daily accumulate physical activity in the field studies for each subject. Many other factors besides individual level of daily physical activity for a healthy lifestyle are cultural, social, and personal.[7] Physical activity intervention had no effect on BMI in children.[8] Data on the inter-relationship between BMI and physical activity are limited. A clear understanding of the effect of physical activity on BMI is lacking. The present study was undertaken to assess the influence of physical activity on maintaining normal grades of BMI and body fat percent among North Indian population.

## MATERIALS AND METHODS

### Study population

Health camp for monitoring of body composition was organized for public by our institution. One thousand two hundred and thirty six urban male visitors volunteered for the study. We have categorized the volunteers participated in the study into two groups: (a) volunteers performing any one form of physical activity and (b) volunteers not doing any physical activity.

Inclusion criteria for volunteers doing physical activity: volunteers should perform yoga exercises or weight-reduction exercises or walking during morning or evening at least three days a week in addition to daily physical activities. Exclusion criteria of volunteers: volunteers with major health complaints like cardiovascular diseases, metabolic disorders, etc. Control population was screened on the basis of not doing any physical activity or exercise (inclusion criteria) other than daily physical activities with no major health problems.

A questionnaire was developed for the study based on International Physical Activity Questionnaire (IPAQ - English version) which has undergone reliability and validity.[9] A pilot study was conducted on 50 volunteers with age group, sex, and occupation similar to study group. This was done to gather data for the reliability and validity of the questions in the questionnaire. The pilot study was able to find out which version of the question resulted in more reliable results. The pilot study also enabled to solicit reactions from respondents on the questionnaire length and parts of questionnaire which respondents felt difficulties in. Substantial revisions were made in the questionnaire based on the pilot study. The questionnaire was filled by the investigator. The selection of volunteers with or without physical activity was determined based on the questionnaire.

Out of total volunteers participated in body composition monitoring, 469 visitors were performing any one form of physical activity and 767 (controls) were not doing any physical activity with similar age group and socioeconomic status. The measurements (height, weight, body fat percent) were taken before lunch and about two hours after light breakfast to avoid diurnal variation of water content in the body. The experimental work of the study was approved by institutional human ethical committee.

### Sampling methods

We used stratified random sampling method for the selection of subjects in three strata among volunteers – school teachers, employees from public sector undertakings, and employees from private sector undertakings. The sample size was calculated considering the overweight prevalence of approximately 10%with precision of 0.025 and 95% confidence limit. It was doubled to minimize the sampling design affect.[10]

### Assessments

#### Body mass index

Height and weight were recorded for each participant to determine their BMI. Height was measured on a tape attached to a wall and rounded down to nearest centimeter. Weight was measured on a body fat monitor HBF 352 (Omron Co., Ltd, Japan) and rounded up to nearest kilogram. Cut-off points according to WHO[11] were used to define the prevalence of overweight.

#### Body fat percent

Body fat was analyzed with Bioelectric Impedance (BI) method. An Omron HBF 352 body fat monitor (Omron Co., Ltd, Japan) was used. BI method was considered reliable and valid to measure resistance in biological tissue.[12–14] A weak electric current is passed between two electrodes placed on different parts of the body and measures the level of resistance in different segments of the body. Body fat percentage was calculated based on the height, age, gender, bodyweight, and electric resistance of the body. Volunteer should stand upright with stretched arms parallel to the floor and the hands gripped for contact to the instruments measuring area. Body fat in percent was calculated using the manufacturers’ equation and digital display was recorded by the investigator on the proforma. Grading of body fat percentage was done based on the earlier reference.[15]

#### Definitions

According to WHO classification (2004), BMI standard were as follows: < 18.5 is underweight, 18.5–24.9 Kg/m^2^ is normal, and 25–29.9 Kg/m^2^ is overweight. Grading of body fat percentage was as follows: < 10% as low, 10–25% as normal (20–25% being border line group was also accommodated as normal for statistical convenience), and 25–30% as high.

### Statistical analysis

Statistical significance of mean values of physical characteristics (age, height, and weight), in two categories was analyzed using student’s ‘*t*’ test for each parameter separately. Prior to analysis, homogeneity of variance and normality of data for each parameter was ascertained. Significance of prevalence rates of personal habits, BMI grades, and body fat percent categories in relation to different grades were tested using Chi-square test. Level of significance was considered to be 5%.

## RESULTS

Table 1 shows physical characteristics, personal habits (smoking and alcoholism), and type of diet among volunteers doing physical activity and not doing physical activity. Forty five percent volunteers from physical activity group and 30% from nonphysical activity group were nonvegetarians. Remaining volunteers were vegetarians in both groups. Seventy percent volunteers of both groups were smokers. Alcoholic consumption among physical activity and nonphysical activity groups was 17.1% and 15.1%, respectively.

**Table 1:** General information of the study population doing physical activity and not doing physical activity

Parameters	NDP (*N* = 767)	DP (*N* = 469)	*t* value
Mean age in years (population %)	36.6 (11.4)	37.4 (10.7)	1.22
Mean height in centimeters (population %)	168.6 (9.1)	170.6 (7.3)	4*
Mean weight in kilograms (population %)	69.7 (13.3)	71.6 (10.9)	2.60*
Smokers (%)	129 (16.8)	79 (16.8)	0
Alcohol consumers (%)	116 (15.1)	80 (17.1)	0.81
Nonvegetarians (%)	231 (30.2)	210 (45.3)	28.8***

NDP - Not doing physical activity, DP - Doing physical activity, *N* - Total number of subjects, *n* - Number of subjects, age, height, and weight represented in mean and SD, Percentage of smokers, alcoholics, vegetarians, nonvegetarians represented in *n* and % and significance given in chi-square, Yr - Year, cm - Centimeter, Kg - Kilogram,

* *P* < 0.05,

*** *P* < 0.001

Table 2 shows grades of BMI among volunteers of the study. Fifty three percent of the physical activity performers had normal BMI compared to 49% nonphysical activity performers. Overweight was observed in 43.3% physical activity performers compared to 44.7% nonphysical activity performers. The difference of prevalence in overweight among subjects doing physical activity and subjects not doing physical activity was found to be nonsignificant.

**Table 2:** Grades of body mass index in study subjects

Type	*N*	*n* (BMI < 18.5%)	*n* (BMI 18.5−24.9%)	*n* (BMI ≥ 25%)	RR
DP	469	15 (3.2)	251 (53.5)	203 (43.3)	1.0
NDP	767	48 (6.2)	376 (49)	343 (44.7)	1.03*

NDP - Not doing physical activity, DP - Doing physical activity, *N* - Total number of study subjects, *n* - Number of subjects, Percentage of DP and NDP for grades of BMI represented in *n* and % and significance given in chi-square,

* *P* > 0.05, RR - Relative risk of overweight (BMI > 25) in NDP compared to DP

Table 3 shows grades of body fat percent among the study subjects. The body fat percent in 52% physical activity performers was in the normal range compared to 48.5% nonphysical activity performers. Low body fat percent was observed in 23.4% physical activity performers compared to 2.7% nonphysical activity performers. High body fat percent was observed in 48.7% nonphysical activity performers compared to 45.8% physical activity performers. However, the percentage differences between the groups were found not to be significant.

**Table 3:** Grades of body fat percent in study subjects

Type	*N*	n (BFP < 10%)	n (BFP 10−25%)	n (BFP ≥ 25%)	RR
DP	469	11 (23.4)	243 (51.8)	215 (45.8)	1.00
NDP	767	21 (2.7)	372 (48.5)	374 (48.7)	1.06

NDP - Not doing physical activity, DP - Doing physical activity, *N* - Total number of study subjects, *n* - number of subjects, BFP - Body fat percent, RR - Relative risk of BFP> 25% in NDP compared to DP

## DISCUSSION

Volunteers (53.5%) performing physical activity maintain normal BMI and body fat percent (51.8%) compared to nonphysical activity performers. A high prevalence of overweight (44.7%) and high body fat percent (48.7%) was observed in nonphysical activity performers compared to physical activity performers in the study. Twenty three point four percent of volunteers had <10% body fat among physical activity performers compared to nonphysical activity performers. Also, the relative risk of overweight and high body fat percent was lower among volunteers doing physical activity than nonphysical activity performers. These observations suggest that physical activity alone cannot maintain BMI and body fat percent, but it can reduce the risk of overweight and high body fat percent in the population. Our study supports the World Health Organization[16] report on preventing and managing the global epidemic of obesity and National Institute of health (NIH) report on the identification, evaluation, and treatment of overweight and obesity.[17] Earlier study by our group has shown that socio-economic status affect body mass index in North Indian adult population[18] These reports show that increased physical activity is important in efforts to lose weight because it increases energy expenditure and plays an integral role in weight maintenance. In addition, increased physical activity may help to reduce body fat and prevent the risk of heart disease.

The variation in duration and frequency of physical activity performance among volunteers was a major limitation of the study. Smoking in 16.8% and alcohol consumption in 15.1% of physical activity performers in the study may have attributed for their overweight and high body fat percent. Overall, the trends in the study show that physical activity alone cannot maintain BMI and body fat percent.
